# Restored T-cell activation mechanisms in human tumour-infiltrating lymphocytes from melanomas and colorectal carcinomas after exposure to interleukin-2

**DOI:** 10.1038/sj.bjc.6600679

**Published:** 2003-01-28

**Authors:** F De Paola, R Ridolfi, A Riccobon, E Flamini, F Barzanti, A M Granato, G L Mordenti, L Medri, P Vitali, D Amadori

**Affiliations:** 1 Department of Medical Oncology, Pierantoni Hospital, Via Forlanini, 34, AUSL-Forli 47100, Italy; 2 Istituto Oncologico Romagnolo, Corso Mazzini 65, Forli 47100, Italy; 3 Department of Pathology, Pierantoni Hospital, AUSL-Forli 47100, Italy

**Keywords:** TIL, tumour immunosuppression, IL-2

## Abstract

We investigated the effects of interleukin-2 (IL-2) exposure on T-cell signal transduction molecules and apoptosis markers in tumour-infiltrating lymphocytes (TIL) isolated from 20 melanoma and 16 colorectal carcinoma metastases and expanded *in vitro* for therapeutic reinfusion. Before IL-2 culture, TIL showed undetectable or very low levels of T-cell receptor (TCR) *ɛ* chain, p56^lck^, Fas ligand (FasL) and Bax expression, while Bcl-2 values were elevated. Cancer cells were characterised by low or absent Fas and Bcl-2 and high Bax expression. Notably, they also expressed FasL. After 41–48 days of IL-2 culture, TCR *ɛ* chain and p56^lck^ expression of TIL rose to median values of approximately 80 and 30% positive cells, respectively (*P*<0.001), FasL expression was detected in 45% cells from melanomas (*P*<0.001) and in 3% from colorectal carcinomas (*P*=0.09), and Bax-positive cells increased from 17.5 to 70% (*P*=0.005). Moreover, TCR *ζ* chain-positive cells were significantly increased from baseline (*P*=0.001), Bcl-2-positive cells dropped from 50 to 1% (*P*=0.007) and perforin content was high, while Fas expression was not significantly modified by IL-2 culture. In conclusion, our data suggest that the degree of immunosuppression in TIL from melanomas and colorectal carcinomas is very high, and the apoptosis markers' repertoire of cancer cells resembles that of immune-privileged tissue. Interleukin-2 culture appears to restore lymphocyte activation mechanisms, resulting in consistent FasL expression and perforin production.

Although the immune system has the ability to recognise and kill tumour cells through its lymphoid effectors, as shown by the findings of both *in vitro* and *in vivo* studies (Topalian and Rosenberg, 1989), it is well known that cancer patients generally have an inadequate antitumour response. Probably several mechanisms underlie the lack of efficient spontaneous immune reactions observed in cancer patients and are responsible for the poor response rates to immunotherapy (Oliver and Nouri, 1992; Sulitzeanu, 1993; Atkins *et al*, 1999). For many years, researchers have focused attention on the effects of tumour-induced immunosuppression on T lymphocytes infiltrating, or associated with, solid tumours (Whiteside, 1999). There is evidence from numerous investigations that tumour-infiltrating lymphocytes (TIL) isolated from a wide range of tumours are functionally impaired, as manifested by decreased proliferative responses and decreased ability to mediate cytotoxicity (Whiteside, 1992; Miescher *et al*, 1998). Abnormalities in signal transduction molecules associated with T-cell receptor (TCR) function have been identified as one of the main mechanisms by which the tumour microenvironment affects immune cells. In particular, reduced expression of TCR-associated *ζ* chain is one of the more consistent findings. Initially discovered in TIL of mice bearing colon carcinoma MCA-38 cells (Mizoguchi *et al*, 1992), abnormalities in ζ chain expression were then isolated in TIL of patients with various types of cancer, including colorectal carcinoma, renal cell carcinoma and metastatic melanoma (Finke *et al*, 1993; Nakagomi *et al*, 1993; Rabinowich *et al*, 1996). It has been shown that decreased or absent expression of the *ζ* chain results in signalling defects in TIL or tumour-associated lymphocytes (TAL), such as altered expression of the tyrosine kinase p56^lck^ and a decreased ability to mobilise intracellular Ca^2+^ in response to activation signals (Mizoguchi *et al*, 1992; Finke *et al*, 1993; Nakagomi *et al*, 1993; Lai *et al*, 1996; Rabinowich *et al*, 1996). Altered *ζ* chain is also associated with impaired cytokine production, as shown by decreased mRNA and protein levels of interleukin 2 (IL-2) and interferon-*γ* (Rabinowich *et al*, 1996) in T cells. It has been suggested that *ζ* chain degradation in TIL and TAL is caused by tumour-induced activation of intracellular peptidases in T cells, and that this process may ultimately lead to a caspase-dependent apoptotic cascade in activated lymphocytes (Rabinowich *et al*, 1998; Whiteside, 1999). The biological significance of *ζ* chain degradation has recently been confirmed by the results of a retrospective study conducted in patients with oral carcinoma, where low or absent *ζ* chain expression in TIL was found to predict poor survival, independently of other factors (Reichert *et al*, 1998).

Another mechanism that may be playing an important role in mediating tumour-induced immunosuppression is the Fas/FasL pathway (Whiteside and Rabinowich, 1998). Recent studies have shown that tumour cells can assume characteristics similar to those of immune-privileged tissues such as the low expression or absence of surface Fas receptor (Fas) and the expression of Fas ligand (FasL) (Hahne *et al*, 1996; Nagata, 1996; O'Connell *et al*, 2001). In addition to other mechanisms (i.e. inhibitory cytokines), tumour FasL surface expression may contribute to T-cell damage and apoptosis (Whiteside and Rabinowich, 1998). However, this hypothesis, formulated on the basis of several authors' independent observations (Whiteside, 1999; Restifo, 2000), has yet to be confirmed.

The finding that tumour-induced degradation of signalling molecules can be ‘reverted’, and proliferative and cytotoxic activities of TIL restored by culture with exogenous cytokines (especially IL-2), at least *in vitro*, has important potential therapeutic implications (Salvadori *et al*, 1993; Rabinowich *et al*, 1996; Zier *et al*, 1996), although data about the *in vivo* effects of cytokines administration on T-cell signalling molecules of cancer patients are still scarce (Farace *et al*, 1994; Rabinowich *et al*, 1996).

In the present study, we carried out a multiparameter analysis of TIL used for therapeutic reinfusion in patients with advanced melanoma and colorectal cancer who had previously undergone metastasectomy. In particular, we evaluated TCR *ζ* and *ɛ* chains, p56^lck^, Bcl-2 and Bax expression, FasL and Fas expression on the surface of TIL from surgically obtained tumour samples, and reassessed the same parameters, with the addition of perforin, after TIL were coincubated with IL-2. In parallel, apoptosis markers in cancer cells of surgically obtained specimens were also examined. The aims of the study were to provide an overview of the parameters involved in the mechanisms of immunosuppression in human TIL from melanomas and colorectal carcinomas cultivated for therapeutic purposes and to assess the *in vitro* effects of exogenous IL-2 on such parameters.

## MATERIALS and METHODS

### Patients

From February 1993 to December 1999, 63 patients with advanced melanoma or colorectal cancer who had undergone metastasectomy were treated with TIL plus IL-2. Clinical results of this trial have been described elsewhere (Ridolfi *et al*, 1998). The clinical–biological study was examined and approved by the Ethics Committee of the Local Health and Social Services (Azienda USL, Forli') in accordance with the ethical standards laid down in the 1964 Declaration of Helsinki. All patients gave their informed written consent to receive treatment.

This biological study was performed in the last 36 consecutive patients, whose median age was 54 years (range 23–70). Patient and tumour characteristics are shown in Table 1

**Table 1:** Patient and tumour characteristics

	**Melanoma**	**Colorectal carcinoma**
*Patient number*	20	16
Male	11	14
Female	9	2
		
Advanced patients^a^	8	3
		
NED patients^b^	12	13
		
*Metastases removed*		
Lymph nodes	13	
Skin	5	
Liver		11
Lung	2	3
Adrenal		1
Peritoneum		1

a Patients with evidence of disease after metastasectomy.

b Patients without evidence of disease after metastasectomy.

.

### TIL culture and expansion

Tissue obtained from metastatic lesions was mechanically fragmented and incubated for 2–16 h in a solution of enzymes containing collagenase (type IV 0.1%), hyaluronidase (type V 0.01%) and DNase (type I 0.00007%). Mononuclear cells were separated according to density gradients. The cell suspension obtained was cultured at a concentration of 1×10^6^ cells ml^−1^ in multiwells using AIM-V medium (Gibco BRL, Grand Island, NY, USA) supplemented with 6000 UI/ml^−1^ recombinant IL-2. Cells were diluted to a concentration of 1×10^6^ ml^−1^ every 3–4 days. The cell culture was transferred to culture bags (Baxter-Fenwal, Deerfield, IL, USA) once the total number of cells had exceeded 0.5–1×10^9^. Phenotyping and immunohistochemical analysis were carried out to check for the presence of residual tumour cells. An aliquot of the cell suspension was cultured without IL-2 to allow tumour cell growth. Suspensions of tumour cells and TIL were cryopreserved in liquid nitrogen in 10% dimethylsulphoxide and 90% foetal calf serum for subsequent studies. When cell numbers had exceeded 1×10^10^, the lymphocytes were concentrated using a CS 3000 Baxter cell separator and reinfused with IL-2 into the patient over 2–3 h. Screening for contaminating microorganisms was performed on the cell suspension 48 h before reinfusion. Tumour-infiltrating lymphocytes in the original tumour specimen are referred to as TIL-1, while lymphocytes at the end of the culture described above are referred to as TIL-2. TIL-2 median culture periods were 41 days (range, 33–62) for melanoma and 48 days (range, 36–74) for colorectal carcinoma.

### Cell line culture

Chronic human myelogenous leukaemia (K562), human Burkitt lymphoma (Daudi), acute human T-cell leukaemia (Jurkat), acute human lymphoblastic leukaemia (MOLT-4) and human promyelocytic leukaemia (HL-60) cell lines were obtained from Istituto Zooprofilattico Sperimentale (Brescia, Italy). All cell lines were maintained in complete RPMI-1640 medium (RPMI-1640 supplemented with 10 mM L-glutamine, 10 *μ*g ml^−1^ streptomycin, 100 U ml^−1^ penicillin (all from Mascia Brunelli, Milan, Italy)) and 10% heat-inactivated foetal calf serum (56°C for 30 min) (Gibco BRL). The above cell lines were used as positive controls for the various monoclonal antibodies (mAbs).

### Monoclonal antibodies

The following panel of mAbs was used: 6B10.2 (anti-CD3 *ζ*, 1 : 150 dilution), UCH-T1 (anti-CD3 *ɛ*, 1 : 125 dilution), 3A5 (anti-p56^lck^, 1 : 100 dilution), C-20 (anti-CD95, 1 : 300 dilution), C-20 (anti-CD95L, 1 : 400 dilution), N-20 (anti-CD95L, 1 : 350 dilution) (all from Santa Cruz Biotechnology, Santa Cruz, CA, USA), 124 (anti-Bcl-2, 1 : 50 dilution), APO-1 (anti-CD95, 1 : 20 dilution), DT-T1 (anti-CD43, 1 : 50 dilution), rabbit anti-human T cell (anti-CD3 *ɛ*, 1 : 50 dilution) (all from Dako Co., Carpinteria, CA, USA), rabbit anti-Bax (1 : 1000 dilution) (PharMingen, San Diego, CA, USA). Each antibody was titrated on positive stabilised cell lines for the parameters evaluated to find the correct dilution needed to detect the specific sites. Two different clones were used for CD95 and CD95L and both produced almost identical results. The results reported in the present study refer only to the use of C-20 (anti-CD95) and N-20 (anti-CD95L) (Smith *et al*, 1998; Mottolese *et al*, 2000).

### Immunocytochemical (ICC) and immunohistochemical (IHC) stainings

Immunocytochemical staining was performed on TIL-2 and IHC staining on TIL-1 and on the tumour cells of the surgically removed specimen. Tumour-infiltrating lymphocyte immunostaining was carried out at the moment of reinfusion on both cytocentrifuged slides and paraffin-embedded tissue derived from the tumours treated to obtain TIL expansion. Tumour-infiltrating lymphocyte cell suspensions were cytocentrifuged at 800 rpm for 10 min on adhesive glass slides using a Shandon centrifuge (Shandon Inc., Pittsburgh, PA, USA). Cytospins were air-dried, fixed with acetone for 10 min and stained with the various antibodies. Sections (5 *μ*m thick) obtained from paraffin blocks of tissue fixed in 10% neutral buffered formalin were mounted on positive-charged slides (Bio-Optica, Milan, Italy) and then deparaffinised.

With the exception of CD43 and Bax, all antigens underwent antigen retrieval (microwaved in 10 mM citrate buffer (pH 6.0) for 15 min at a medium–high setting). Endogenous peroxidase was inactivated with 3% hydrogen peroxide, and aspecific sites were inactivated with bovine serum albumin (BDH Laboratory Supplies, Poole, Dorset, UK) in phosphate-buffered saline (PBS concentration NaCl 130 mM; Na_2_HPO_4_, 12H_2_O 6 mM; KH_2_PO_4_ 1.55 mM) in both histological sections and cytospins. The samples were incubated at room temperature for 1 h with different antibodies. Staining was performed according to the manufacturer's instructions using the LSAB^+^ peroxidase complex kit (Dako Co.). Immunoreactivity was visualised with 2,2′-azino-bis(3-ethylbenthiazoline-6-sulphonic acid) (Dako Co.) and the samples were counterstained briefly with haemotoxylin. Positive controls consisting of tonsillar sections, cytospins and tumour cell line paraffin blocks were stained with the different antibodies. Immune serum was omitted in negative controls. Total lymphocytes were identified by the expression of CD43 (Borche *et al*, 1987), and the proportion of CD43-positive cells was always higher than 70% both for TIL-1 and TIL-2. CD43 found in TIL-1 and TIL-2 had median values of about 90%, thus confirming that the tests were performed mainly on lymphocytes.

In previous experiments, we tested the sensitivity of the IHC method used in the study by comparing the results obtained with frozen tissue and paraffin-embedded tissue from 20 cases of melanoma or renal carcinoma using different dilutions of antibodies. Similar results were observed with the two processing techniques at the dilutions used in the present study. Furthermore, in cell suspensions from the tumour cell lines, we compared the results obtained by using cytocentrifuged slides *vs* paraffin-embedded cell blocks. Again, the results were similar (Ridolfi *et al*, unpublished data).

### Identification and expression of perforin

TIL perforin expression was determined at the moment of reinfusion. Lymphocytes (1×10^6^) were fixed with 2% cold paraformaldehyde solution (Sigma, St Louis, MO, USA) and resuspended in PBS containing 0.2% Tween-20 to permeabilise cells. The lymphocytes were then washed and incubated with δ G9 monoclonal antibody (anti-human perforin) conjugated with fluorescein isothiocyanate (FITC), resuspended in 1% paraformaldehyde and analysed. Negative control cells were incubated only with FITC-conjugated goat anti-mouse Ig. Flow cytometry analysis was performed with a FACStar Plus (Becton Dickinson, San Jose, CA, USA) flow cytometer, calibrated for fluorescence intensity measurements using FITC-labelled microbead standards (fluorescence-activated cell sorting) with assigned MESF values (molecules of equivalent soluble fluorochrome). Lymphocytes were selected using polygonal windows that excluded cell aggregates, debris and morphologically aberrant elements. 5×10^3^ cells were analysed for each sample.

### FACS analysis

TIL were phenotyped with monoclonal antibodies recognising CD3, CD4, CD8, CD16, CD56, CD19, CD25, CD71 and HLA-DR after *in vitro* expansion. Lymphocytes were suspended in Hank's balanced salt solution (without phenol red), incubated with each monoclonal antibody for 20 min in the dark and then washed.

### Cytotoxicity analysis 

A cytotoxic assay was performed on TIL-2 from a small number of melanoma cases (7/20) using the ^51^Cr release assay just before patient reinfusion. Target cells included fresh (or cryopreserved) autologous or allogenic tumour cells, and K562 cells. TIL cytolytic activity was determined by plating 1000 target cells labelled with ^51^Cr in V-bottom microtitre plates. Effector cells were then added at various concentrations to achieve effector:target ratios of 100 : 1, 50 : 1, 25 : 1 and 12 : 1. Microtitre plates were incubated in a humidified incubator with 5% CO_2_ for 4 h, then 100 *μ*lwell^−1^ of supernatant was removed and their radioactivity counted in a scintillator. Lysis percentage was calculated according to the following formula:

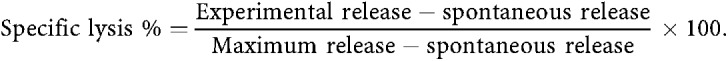
.

### Statistical analysis

The Wilcoxon pairwise rank tests were used to analyse the variations in the values of the biological parameters observed in TIL-1 and TIL-2. Spearman's nonparametric correlation coefficient was used to investigate the relation between the different biomarkers. All *P*-values were based on two-sided testing (threshold value=0.05), and statistical analyses were carried out with the SPSS package.

The study was conducted with a purely exploratory intent. Multiple tests were performed on this limited case series, increasing the risk of finding statistical differences that were due to chance. As no statistical correction was made, the *P*-value must be interpreted with caution.

## RESULTS

The expression of T-cell transduction signals and apoptosis markers was analysed in TIL before (TIL-1) and after (TIL-2) *in vitro* culture with IL-2 in 20 patients with advanced melanoma and in 16 with advanced colorectal cancer (Table 1).

### Signal transduction molecules

**Figure 1:**
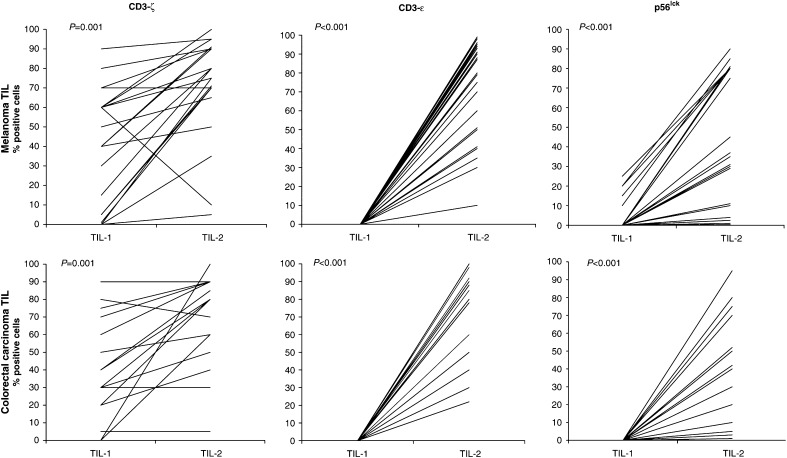
Signal transduction molecules. The percentage of positive lymphocytes was evaluated before (TIL-1) and after (TIL-2) culture with IL-2. TCR, *ζ* and *ɛ* chains and p56^lck^ expression was evaluated in TIL-1 by IHC staining on paraffin-embedded surgically removed specimens. The same molecules were evaluated in TIL-2 by IHC staining on cytocentrifuged slides. The assays were performed on 20 melanoma and 16 colon carcinoma samples.

The data regarding signal transduction activating molecules in TIL-1 and TIL-2 are shown in Figure 1

. We observed an increase in the expression of *ɛ* and *ζ* chains and p56^lck^ after culture with IL-2. In particular, p56^lck^ and the ɛ chain had a very low or null expression in TIL-1, which increased up to 100% positive cells after exposure to IL-2 (TIL-2). T-cell receptor *ζ* chain expression was significantly higher after culture, although some patients (those with high pretreatment values) showed no evidence of an increase at the end of culture. The low level of expression of T-cell signal transduction molecules in TIL-1 and the increase produced by *in vitro* culture in the presence of IL-2 were observed in both melanoma and colorectal carcinoma patients.

### Apoptosis-related molecules

**Figure 2:**
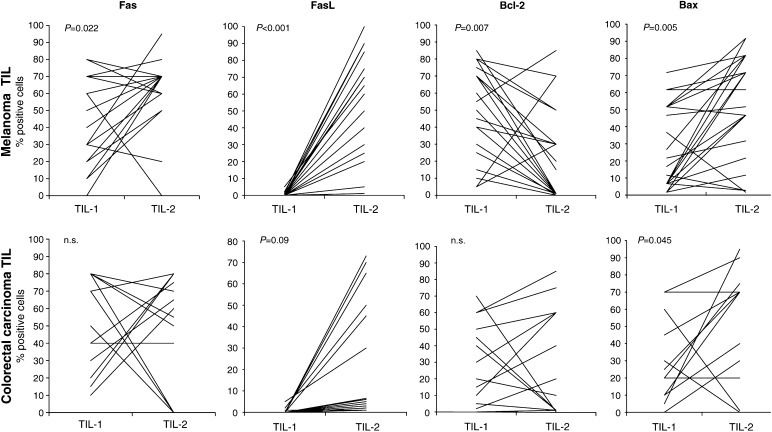
Apoptosis-related molecules. The percentage of positive lymphocytes was evaluated before (TIL-1) and after (TIL-2) culture with IL-2. Fas receptor, FasL, Bcl-2 and Bax expression was evaluated in TIL-1 by IHC staining on paraffin-embedded, surgically removed specimens. The same molecules were evaluated in TIL-2 by IHC staining on cytocentrifuged slides. The assays were performed on 20 melanoma and 16 colorectal carcinoma samples.

The data regarding apoptosis markers in TIL-1 and TIL-2 are shown in Figure 2

. IL-2 induced a considerable increase in FasL expression, which was absent in TIL-1, whereas Fas expression was not significantly modified. Bcl-2 and Bax levels showed, as expected, inverse changes: the median percentage of Bcl-2-positive cells dropped from 50 to 1 (*P*=0.007) and, in parallel, median Bax-positive cell percentages rose from 17.5 to 70 (*P*=0.005).

Fas receptor, FasL, Bcl-2 and Bax expression determined in tumour cells from surgically removed metastases is shown in Table 2

**Table 2:** Apoptosis markers in tumour cells: percentage of positive cells

	**Melanoma**		**Colorectal carcinoma**	
	**Median (%)**	**Range**	**Median (%)**	**Range**
				
Fas	1.5	0–25	0.5	0–15
FasL	35	0–80	22.5	0–80
Bcl-2	5	0–80	0	0–65
Bax	80	0–90	70	5–90

Apoptosis markers were evaluated by immunohistochemical staining on the tumour cells present in paraffin-embedded surgically removed specimens.

. Median values were similar for the two tumour types and showed low or absent Fas and Bcl-2 expression, appreciable levels of FasL and very high levels of Bax expression. However, there was a great variation in apoptosis marker levels among individual specimens.

### TIL activation determinants

The extent of TIL activation after culture with IL-2 was analysed by testing the surface determinants and the level of perforin.

TIL-2 phenotypes, measured by FACS analysis, showed a similar percentage of positive cells for melanoma and colorectal carcinoma with regard to CD4, CD8, CD56 and HLA-DR. As reported in the literature (Rosenberg *et al*, 1994; Goedegebuure *et al*, 1995), the TIL phenotype consisted mainly of CD8 (median value 84%: range 14–98) and HLA-DR (median value 66%: range 13–98) positive cells ( Table 3

**Table 3:** Phenotypes of TIL-2 from melanoma and colorectal carcinoma metastases: percentage of positive cells

	**Melanoma**		**Colorectal carcinoma**	
	**Median (%)**	**Range**	**Median (%)**	**Range**
CD4	1.5	0–68	6	0–96
CD8	84	14–98	73.5	0–99
CD56	16	2–75	8	2–35
HLA-DR	66	13–98	61	25–99

).

Perforin levels in the supernatant were determined at median culture periods of 41 (range 33–62) and 48 (range 36–74) days for TIL from melanomas and colorectal carcinomas, respectively, and were elevated in both tumour types, especially in TIL from colorectal carcinomas (median MESF 22,118; range 10 000–81 000).

### Cytotoxicity

In the few TIL-2 melanoma cases (7/20) in which cytotoxicity was assessed, a low reactivity against K562 cells was generally found, while the reactivity against autologous melanoma cells was slightly higher. It remained, however, at around 20% of lysis ( Table 4

**Table 4:** TIL-2 cytolytic assay (% of lysis) in seven patients with melanoma

**Patient**	**K562 cells**	**Allogenic melanoma cells**	**Autologous melanoma cells**
1	1	4	16
2	2	20	21
3	2	3	–
4	12	3	12
5	2	2	18
6	28	15	29
7	7	1	15

Ratio effector : target=25 : 1

).

### Statistical correlations among different parameters

Statistical analysis was performed using Spearman's correlation coefficient to investigate the relation between the different TIL biomarkers before and after exposure to IL-2. In Table 5

**Table 5:** Relationship between the different biomarkers using Spearman's correlation coefficient (*r*)

	**Melanoma (20 cases)**		**Colorectal carcinoma (16 cases)**	
	***r***	***P***	***r***	***P***
*Before culture*				
TIL Fas *vs* tumour FasL		0.51	<0.05	
TIL Fas *vs* tumour Bax	−0.49	<0.05	−0.45	<0.05
				
*TIL after culture*				
Fas *vs* *ζ* chain		0.63	<0.01	
Fas *vs* p56^lck^	0.57	<0.01	0.69	<0.01
				
FasL *vs* *ζ* chain	0.59	<0.01		
FasL *vs* *ɛ* chain	0.61	<0.01	0.49	<0.05
FasL *vs* p56^lck^	0.57	<0.01	0.52	<0.05
				
Perforin *vs* *ζ* chain	0.64	<0.01		
Perforin *vs* p56^lck^ a	0.79	<0.01		

a 13 cases.

Spearman's correlation coefficient was calculated for each different TIL and tumour cell biomarker. Only statistically significant correlations are shown.

only statistically significant correlations are shown. In brief, before IL-2 exposure, there was a negative correlation between Fas and Bax expression in TIL from both melanoma and colon carcinoma, and a positive correlation between Fas and FasL only in TIL from colorectal carcinoma. Following IL-2 exposure, a far larger number of significant correlations were observed for the TIL from both tumour types. Fas expression was positively correlated with p56^lck^ in melanoma TIL, and with p56^lck^ and the *ζ* chain in colorectal TIL. Fas ligand was positively correlated with the *ζ* and *ɛ* chains and p56^lck^ in melanoma TIL, and only with the *ɛ* chain and p56^lck^ in colorectal carcinoma TIL. Moreover, a significant positive correlation was observed between perforin expression and p56^lck^, and perforin and the *ζ* chain in melanoma TIL.

## DISCUSSION

Our observations of TCR-associated signalling molecules and apoptosis markers in TIL obtained from melanoma and colorectal carcinoma metastases support the hypothesis that lymphocytes at the tumour site manifest a high degree of immunosuppression, as shown by absent ɛ chain or p56^lck^ expression, very low *ζ* chain and Fas expression, and, most importantly, extremely low FasL expression. As for the tumour cells, they also had very low Fas but, in contrast, showed a relatively high level of FasL expression. The scenario would therefore seem to be one of strong immunosuppression in TIL conditioned by tumour cells that appear to be very similar to immune-privileged tissue. Abnormalities in TCR-associated molecules, especially *ζ* chain and p56^lck^, have been consistently reported in several investigations carried out on TIL or TAL obtained from cancer patients (Finke *et al*, 1993; Nakagomi *et al*, 1993; Lai *et al*, 1996; Rabinowich *et al*, 1996), as well as tumour-bearing mice (Mizoguchi *et al*, 1992). The possibility has been suggested that these signalling defects may be artefacts because of processing techniques. However, in our study (as in other investigations), such abnormalities were detected *in situ* and would, therefore, be unlikely to be caused by tissue processing.

Our results with IL-2-cultured TIL are consistent with the hypothesis of an IL-2-induced modulation of the markers involved in activation mechanisms, manifesting as a restoration of the *ɛ* and *ζ* chains and, to a lesser degree, of p56^lck^ expression, an increase in FasL expression and an elevated perforin production. The results of the cytotoxic assay, although limited to a small number of cases, appear to support the notion that IL-2-stimulated TIL actually possess a specific cytotoxic activity. Similar patterns of restored activation molecules after IL-2 culture were observed in melanoma and colorectal carcinoma TIL.

After IL-2 culture in melanoma TIL, high levels of Fas and Bax and low Bcl-2 expression were observed. These findings suggest that IL-2 is an important growth and survival factor for T lymphocytes and that it also sensitises these cells to Fas-mediated cell death. The different patterns of Fas and Bcl-2 expression in TIL-2 from colon carcinomas and melanomas could be attributed to different homeostatic conditions in rapidly expanding cell cultures since these two markers are apoptosis related (Van Parijs *et al*, 1999). However, it should be highlighted that tumour Fas and FasL, and Bcl-2 and Bax systems were similar in the two tumour types. In particular, we observed a low expression of Bcl-2 proto-oncogene and a high expression of Bax, in agreement with the literature data (Tang *et al*, 1998). The role of Bcl-2 in malignant transformations is still controversial (Tron *et al*, 1995; Selzer *et al*, 1998), whereas there is evidence to suggest that Bcl-2 expression could be an indicator of lymph node and distant metastases (Grover and Wilson, 1996; Hernberg *et al*, 1998). There are conflicting opinions about the role of tumour FasL in mediating apoptosis of immune cells and counterattacking the host's defence systems (Rivoltini *et al*, 1998; Chappell *et al*, 1999; Restifo and Rosenberg, 1999; O'Connell *et al*, 2001). In a recent review, Whiteside and Rabinovich suggest that the tumour may directly induce post-translational modifications of signal-transducing proteins, such as the *ζ* chain, in T lymphocytes, and that these processes may be a part of the apoptotic cascade initiated in T cells by contact with the tumour cells. They also suggest that both degradation of signalling molecules and apoptosis of T cells may be mediated by the Fas/FasL pathway (Whiteside and Rabinovich, 1998).

In conclusion, the findings of our investigation, in agreement with accumulated evidence from numerous studies, lend support to the hypothesis that the tumour microenvironment induces immunosuppression of infiltrating lymphocytes by downregulating signalling molecules and apoptosis markers. Such alterations, however, can be reverted by removal of TIL from the tumour site and coincubation with IL-2. This ‘rescue’ from local immunosuppressive mechanisms appears to be crucial if the cytotoxic potential of TIL is to be exploited for therapeutic purposes.
